# Gene Expression Profiling and Identification of Resistance Genes to *Aspergillus flavus* Infection in Peanut through EST and Microarray Strategies

**DOI:** 10.3390/toxins3070737

**Published:** 2011-06-24

**Authors:** Baozhu Guo, Natalie D. Fedorova, Xiaoping Chen, Chun-Hua Wan, Wei Wang, William C. Nierman, Deepak Bhatnagar, Jiujiang Yu

**Affiliations:** 1 Crop Protection and Management Research Unit, U.S. Department of Agriculture-Agricultural Research Service, Tifton, GA 31794, USA; Email: Baozhu.guo@ars.usda.gov; 2 The J Craig Venter Institute, Rockville, MD 20850-3343, USA; Email: nfedorova@jcvi.org (N.D.F.); cwan@jcvi.org (C.-H.W.); wwang@jcvi.org (W.W.); wnierman@jcvi.org (W.C.N.); 3 Department of Plant Pathology, The University of Georgia, Tifton, GA 31793-0748, USA; Email: xpchen1011@gmail.com; 4 Crops Research Institute, Guangdong Academy of Agricultural Sciences, Guangzhou 510640, China; 5 Department of Biochemistry and Molecular Biology, The George Washington University School of Medicine, Washington, DC 20037, USA; 6 Southern Regional Research Center, U.S. Department of Agriculture-Agricultural Research Service, New Orleans, LA 70124, USA; Email: deepak.bhatnagar@ars.usda.gov

**Keywords:** EST, microarray, gene profiling, peanut-fungus interaction, resistance genes, *Aspergillus flavus*, *A. parasiticus*, metarep

## Abstract

*Aspergillus flavus* and *A. parasiticus* infect peanut seeds and produce aflatoxins, which are associated with various diseases in domestic animals and humans throughout the world. The most cost-effective strategy to minimize aflatoxin contamination involves the development of peanut cultivars that are resistant to fungal infection and/or aflatoxin production. To identify peanut *Aspergillus*-interactive and peanut *Aspergillus*-resistance genes, we carried out a large scale peanut Expressed Sequence Tag (EST) project which we used to construct a peanut glass slide oligonucleotide microarray. The fabricated microarray represents over 40% of the protein coding genes in the peanut genome. For expression profiling, resistant and susceptible peanut cultivars were infected with a mixture of *Aspergillus* *flavus* and *parasiticus* spores. The subsequent microarray analysis identified 62 genes in resistant cultivars that were up-expressed in response to *Aspergillus* infection. In addition, we identified 22 putative *Aspergillus*-resistance genes that were constitutively up-expressed in the resistant cultivar in comparison to the susceptible cultivar. Some of these genes were homologous to peanut, corn, and soybean genes that were previously shown to confer resistance to fungal infection. This study is a first step towards a comprehensive genome-scale platform for developing *Aspergillus*-resistant peanut cultivars through targeted marker-assisted breeding and genetic engineering.

## 1. Introduction

Peanut (*Arachis hypogaea* L.) has been an important food and oil crop. Peanut contains not only a high percentage of oil (about 50%) but also contains a high quality unsaturated fatty acid (oleic acid). These features confer superior oxidative stability for food products without further processing. Peanut oil is also low in saturated fat and rich in resveratrol, antioxidants, and other nutriceuticals, which may contribute to cardiovascular health. Currently, peanut is grown world-wide, predominantly in Asia, Africa, and North Americas, with about 21 million hectares under cultivation. World peanut production occupies an important role in the world economy with an estimated production value of about $35 billion.

Research on the peanut genome is at an early stage. Major crop improvement emphasis is focused on using elite genetic stocks, cultural management, and disease and pest control measures to improve productivity and quality. Traditionally cultivar improvement has been limited by conventional breeding and selection strategies [1]. High throughput technologies such as whole genome and transcriptome sequencing and microarray analysis hold promise to greatly facilitate this process. To meet the needs of the peanut industry, the international research community developed the International Peanut Genomics Initiative to coordinate sequencing the complete peanut genome (http://www.peanutbioscience.com/peanutgenomeinitiative.html) [2,3]. Peanut is a polyploid organism with a large genome size (2.8 Gb), which makes whole genome sequencing prohibitively expensive. Furthermore, due to its polyploid nature, assembly, annotation, and analysis of the genome will be a very challenging task. Thus, alternative approaches such as Expressed Sequence Tag (EST) sequencing have been implemented to advance the understanding of the genome at a manageable cost. 

Several research institutes have undertaken low to middle scale peanut Expressed Sequence Tag (EST) projects [4,5,6]. As early as 2005, Luo *et al*. [6] released the first batch of EST sequences from two cultivated peanut lines, which were later used to design the first peanut microarray [7,8]. Subsequently, our research group at the USDA reported a total 41,568 ESTs derived from Tifrunners and the breeding line GT-C20 [4,5]. Another group in Belgium generated 4847 ESTs from peanut mixed stages infected with the migratory peanut pod nematode [9]. A group at the University of Florida used suppression subtractive hybridization to identify differentially expressed ESTs from RKN-challenged root tissues in nematode-resistant and -susceptible peanut cultivars [10]. Lately, the Shandong Academy of Agricultural Sciences, China, has started a large scale EST project and has provided 17,000 expressed sequence tags (ESTs) [11].

With the increased awareness of aflatoxin contamination in peanut [2], the presence of aflatoxin in peanut products has become a serious food safety concern. It is a major financial concern to the peanut industry as more regulatory import measures take effect worldwide. Aflatoxin contamination in pre-harvested peanuts is caused by the infection of the *Aspergillis* species, mainly *A. flavus* and *A. parasiticus*. Understanding peanut-fungus interactions during the growth of both the peanut crop and the fungus is necessary to develop effective strategies to reduce or eliminate aflatoxin contamination of pre- and post-harvest peanut crop. Currently, peanut cultivars that are resistant to *A. flavus* and *A. parasiticus* infection are rare, and little is known about the molecular mechanisms that confer such resistance. 

To gain a better understanding of these mechanisms, the USDA has initiated the peanut genome program [2]. We recently [12] developed and tested the utility of the first large-scale peanut microarray, investigating the gene expression in different peanut tissues such as pod, leaf, stem, root, and peg tissues. The study identified 108 putatively pod-specific/abundant genes [12]. Subsequently, as part of U.S. Peanut Genome Initiative supported by U.S. Industry and Peanut Growers, our group developed a large scale peanut EST project [2,4,13] for the cultivated peanut and provided the genomic resources for use in marker development and gene discovery. Here we report the development of a peanut microarray based on these EST sequences as well as other publicly available peanut EST sequences down-loaded from dbEST database (NCBI, http://www.ncbi.nlm.nih.gov/) [14]. We employed this array in gene expression profiling experiments to identify candidate genes that confer resistance to *Aspergillus* infection due to up-expression in response to fungal infection using a resistant peanut line *vs.* a susceptible line.

## 2. Materials and Methods

### 2.1. Peanut Lines Used

Two peanut lines (cultivars) have been used in this experiment: Tifrunner and GT-C20, hereafter referred as C20. “Tifrunner” (TF) is a runner market-type peanut (*Arachis hypogaea* L. *subsp. hypogaea var. hypogaea*) cultivar with a high level of resistance to Tomato Spotted Wilt Virus (TSWV), moderate resistance to early (*Cercospora arachidicola*) and late leaf spot (*Cercosporidium personatum*), but it is a late maturity cultivar [15]. This cultivar is considered susceptible to *Aspergillus* infection in the field. “GT-C20” is a Spanish-type breeding line and highly susceptible to TSWV and leaf spots but resistant to aflatoxin contamination [16].

### 2.2. Peanut Inoculation by *Aspergillus* during Growth

Both resistant and susceptible peanut cultivars were subjected to infection with a mixture of *A. flavus* and *A. parasiticus* spores 60 days after planting (DAP). In order to mimic peanut field fungal population, *A. parasiticus* NRRL 2999 and *A. flavus* NRRL 3357 were used for inoculation because they are pre-dominant fungal strains in our peanut field. Peanut immature kernels were harvested 30 days after inoculation. Total RNAs were isolated from these immature kernel seeds. Poly-A mRNAs were prepared from the total RNAs immediately prior to cDNA library construction.

### 2.3. Expressed Sequence Tags and Sequencing

Tissue collection, RNA isolation, cDNA library construction and sequencing were done at USDA-ARS, Crop Protection and Management Research Unit at Tifton, Georgia and US Horticultural Laboratory Genomics Research Center at Ft. Pierce, Florida. The peanut plant materials used for RNA extraction were grown in the field and inoculated at mid-bloom (60 DAP). Drought stress was imposed during the final 40 days before harvest through the use of rain-out shelters. Immature pods at the R5 (beginning seed), R6 (full seed) and R7 (beginning maturity) stages from “GT-C20” and “Tifrunner” were collected, frozen in liquid nitrogen, and stored at −80 °C until RNA extraction. Leaf tissues were collected at 100 DAP under the natural occurrence of spotted wilt and leaf spot diseases of peanut genotypes, Tifrunner, GT-C20 and A13 [6,7]. Tissues were frozen in liquid nitrogen and stored at −80 °C until RNA extraction by Trizol extraction. Tifrunner is resistant to TSWV and leaf spots, but susceptible to *Aspergillus flavus*. GT-C20 is susceptible to TSWV and leaf spots but resistant to *A. flavus*, and A13 (NCV11 × AR4) is moderately resistant to TSWV and leaf spots, and resistant to *A. flavus* infection [17].

EST libraries were constructed using the pBluescript^®^ II XR cDNA Library Construction Kit (Stratagene, La Jolla, California, Catalog). Briefly, directional cDNA synthesis was made by attaching 5' EcoRI and 3' XhoI adaptors (oligo dT XhoI primer). After digesting with EcoRI and XhoI restriction enzymes, the cDNA inserts were ligated into the multicloning sites of pBluescript II SK (+) plasmid vector. The cDNAs in the pBluescript vector were sequenced using universal primers (5' T3 primer). Single pass, unidirectional (5' end) sequencing was performed using ABI 3730xl Genetic analyzer (Applied Biosystems) with the ABI Prism BigDye terminator cycle sequencing kit (Foster City, CA). Base calling was made using Phred and Trace Tuner (Paracel, Pasadena, CA, USA). The sequencing, sequence cleaning, end trimming, and assembly processing were performed in the Laboratory for Genomics and Bioinformatics, University of Georgia. 

### 2.4. Oligo Microarray Design

The printed oligonucleotide sequences and the array platform description can be found at the NCBI GEO database (accession GPL13178). Briefly, oligonucleotides ranging from 60 to 70 mer were designed at the J. Craig Venter Institute (JCVI) and synthesized by Sigma-Aldrich (Saint Louis, MO). The total number of oligonucleotides spotted on the microarray was 6932, which represented 6932 peanut unigenes. They were spotted to Corning ultraGAPs glass slides with 3 replications of each oligonucleotide at different locations on the slide. With flip-dye hybridizations, the array platform generates 3 technical replications per hybridization. 

### 2.5. Microarray Experiment Design, Hybridization and Analysis

Two factors were varied in the experimental design: peanut cultivars (TF and C20) and *Aspergillus* exposure. Combinations of these two factors allowed for four hybridization probe pairs for competitive hybridization as follows:

- C20Y *vs.* TFY (GT-C20 infected *vs.* Tifrunner infected)
- C20Y *vs.* C20N (GT-C20 infected *vs.* not infected)
- TFY *vs.* TFN (Tifrunner infected *vs.* not infected)
- C20N *vs.* TFN (GT-C20 not infected *vs.* Tifrunner not infected)

The four samples were analyzed with four hybridizations each with a flip-dye control and three in-slide replicates as described (GEO records: GSM684493, GSM684512, and GSM684513). 

### 2.6. Data Processing for EST and Microarray Analysis

Sequencing trace files from the cDNA peanut library were processed following the JCVI Sanger pipeline, which trims off vector and adaptor sequences and removes low-quality bases. Sequences sharing overlapping regions of greater than 94% identity over 40 or more continuous bases were assembled at high stringency using the CAP3 program and Paracel Transcript Assembler [18]; version 2.6.2, (http://www.paracel.com) [19] with modifications by the JCVI bioinformatics team. Overlaps based exclusively on low-complexity regions were excluded. 

Hybridized slides were scanned using the standard protocol (see GEO records: GSM684493, GSM684512, and GSM684513 for details). All calculated gene expression ratios were log_2_-transformed and analyzed using MeV (http://www.tm4.org/mev.html) [20,21,22].

A gene was considered to be expressed if it had a positive expression value associated with it. Log_2_ ratios were used to measure relative changes in expression level between two growth conditions. Genes were considered differentially expressed if the corresponding log_2_ ratios were greater than 2. Gene Ontology (GO), enzyme classification (EC), and PFAM term enrichment analysis was performed using METAREP, an online annotation presentation tool developed at the JCVI (http://www.jcvi.org/metarep/dashboard/index) [23].

## 3. Results and Discussion

### 3.1. Summary Classification of Expressed Sequence Tags(EST)

A total of over 11,141 ESTs were assembled from over 100,000 Sanger reads generated in this study. Additional 2738 EST sequences were downloaded from the NCBI dbEST database including those sequences submitted by Shandong Academy of Agricultural Sciences. From this dataset, 13,879 unique ESTs (unigenes) have been assembled and annotated. The average GC content of these ESTs is 42.6% with the minimum GC of 15.8% and maximum GC of 72.5%. It is estimated that the 2.8-Gb peanut genome hosts 25,000–35,000 protein-coding genes, therefore 13,879 ESTs represent over 40% of these genes. BLASTp search against the NCBI NR database showed that 1761 ESTs (12.7%) can be assigned a putative function based on sequence similarity to previously characterized proteins. However 87.3% of the ESTs (12,118 ESTs) did not have significant hits in the database and were annotated as ‘hypothetical”. Major functional categories represented in this EST set are listed in Table 1. The EST sequence data have been submitted to the NCBI EST database (ES702769 to ES724546 and ES751523 to ES768453). 

**Table 1:** Classification of identified genes in peanut.

Category of Genes	Number of Genes
Hypothetical proteins	12,118
Ribosomal protein	131
Lopprotein	91
Cupin	54
Ribulose bisphophate carboxylase	36
Oleisin	33
Conglutin	32
Photosystem I and II	29
Protease inhibitor/seed storage protein	28
Core histone	25
Ara H8 allergen/alergen	25
Ubiquitin-conjugating enzyme	23
Peptidases	22
Epoxide hydrolase	19
Ras family protein	16
Glutathionine S-transferase	16
Zinc figure protein	14
Seed maturation protein	13
NAD/NADH dehydrogenase	12
Mem brane protein	12
Hsp20	11
Peroxidase	10
14-3-3 protein	10
Universal stress protein	9
Oxidoreductase	9
HMG(high mobility group) box	8
Protein kinase	6
Polygalacturonase	4
Other	1063

### 3.2. Identification of Resistant Genes to *Aspergillus* Infection Using Microarray Expression Data

A 6932 gene-element oligonucleotide microarray was designed according to the 13,879 EST sequence information data set. Four microarray hybridizations were performed. We compared resistant peanut line, GT-C20, and susceptible peanut line, Tifrunner, under *Aspergillus* infected and non-infected conditions (C20Y *vs.* TFY; C20Y *vs.* C20N; C20N *vs.* TFN and TFY *vs.* TFN). The gene expression level is reported as log_2_ ratios of relative intensity. Among the 6932 genes whose RNA level was detected by the microarray, there were 401 genes that showed significant changes in gene expression level between resistant and susceptible peanut lines under infected and non-infected conditions. For each specific microarray hybridization, the number of up (log_2_ ≥ 1.5) and down (log_2_ ≤ −1.5) expressed genes are summarized in Table 2. It is interesting to find that there were a large number of genes in the resistant peanut line GT-C20 either highly or moderately up-expressed. Without *Aspergillus* infection (C20N *vs.* TFN), there were 9 and 31 genes in GT-C20 that scored as highly and moderately up-expressed compared with the susceptible line Tifrunner (C20N *vs.* TFN). With *Aspergillus* infection, the highly and moderately up-expressed genes were 25 and 40 respectively compared to the same strain without infection (C20Y *vs.* C20N). More interestingly, the resistant line, GT-C20, demonstrated a greater response to *Aspergillus* infection than the susceptible line Tifrunner (C20Y *vs.* TFY). The highly and moderately up-expressed genes were 52 and 126 respectively (C20Y *vs.* TFY). On the other hand, the susceptible line Tifrunner showed almost no response to *Aspergillus* infection (TFY *vs.* TFN). When under challenge by *Aspergillus* species, only one gene showed moderate up expression and four genes showed moderate down expression. 

**Table 2:** Statistics of differentially expressed genes among 6932 expressed genes in peanut as detected by microarray.

	Differential Expression			
Hybridizations	Up-high (Log_2_ ≥ 2)	Up-mod (Log_2_ ≥ 1.5 &< 2)	Down-high (Log_2_ ≤ −2)	Down-mod (Log_2 _≤ −1.5 & > −2)
C20Y *vs.* TFY	52	126	51	99
C20Y *vs.* C20N	25	40	9	38
C20N *vs.* TFN	9	31	3	19
TFY *vs.* TFN	0	1	0	4

Table 3 shows the 62 genes among the 178 up-expressed genes shown in Table 2 column 1 and column 2 (52 up-high and 126 up-mod) that were consistently highly up-expressed in response to *Aspergillus* infection in GT-C20 across two experiments (C20Y *vs.* TFY; C20Y *vs.* C20N) with expression levels significantly elevated (log_2_ ≥ 1.5). While under non-infection condition, the expression levels are about the same as the susceptible line (C20N *vs.* TFN) (Table 3). Unfortunately, among the 62 expression elevated genes, only 8 genes were assigned biological functions based on their homologies to the corresponding genes in the database. The remaining 54 genes were classified as hypothetical proteins with no homologs in the existing database. From the consolidated data, we identified 22 genes in the resistant line (GT-C20) that were constitutively up-expressed compared with the susceptible line (Tifrunner) under infected (Table 4, C20Y *vs.* TFY, log_2_ values ≥ 1.5) and non-infected conditions (Table 4, C20N *vs.* TFN, log_2_ values ≥ 1.0). Among the 22 genes, 5 genes showed slightly up-expression in response to *Aspergillus* infection compared with non-infection conditions (C20Y *vs.* C20N). Table 5 lists 42 genes in the resistant line GT-C20 that were consistently highly down-expressed in response to *Aspergillus* infection. Table 6 lists 24 genes in the resistant line GT-C20 that were constitutively down-expressed in the absence of infection or slightly down-expressed in response to *Aspergillus* infection (C20Y *vs.* TFY).

**Table 3:** Peanut genes consistently highly expressed in response to fungal infection.

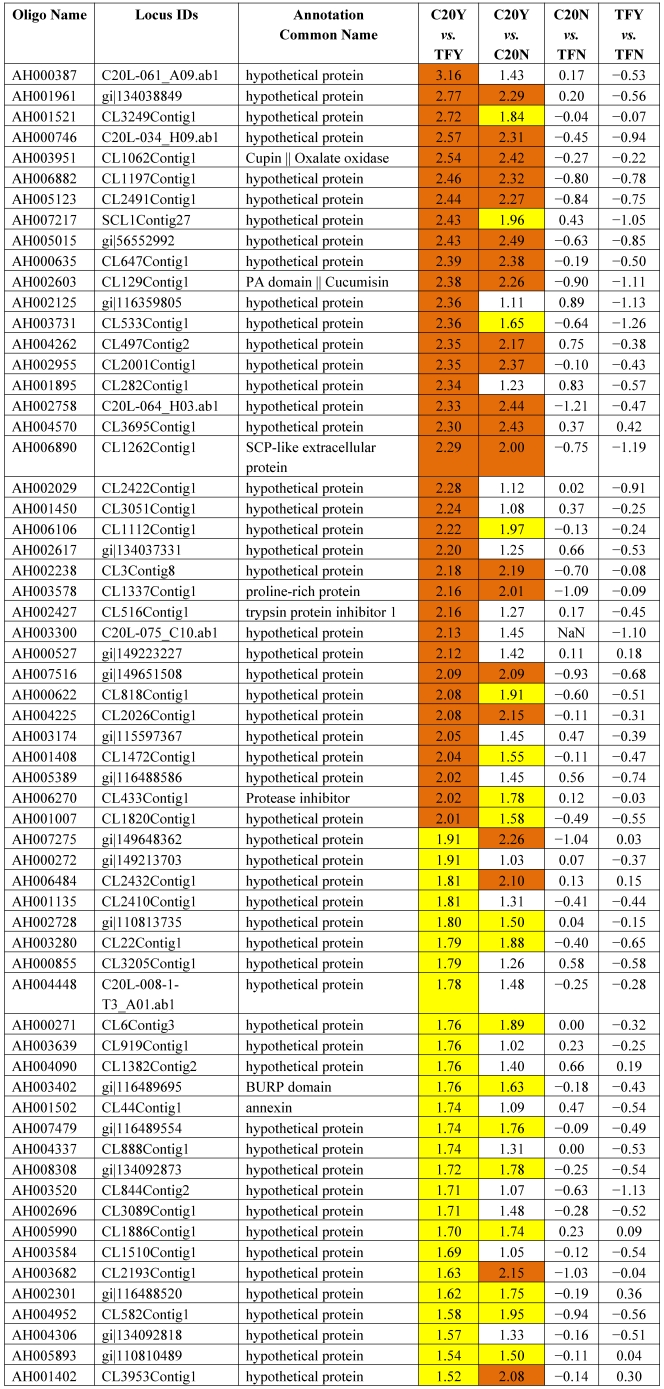

Note: The values are log_2_ ratios. For example, C20Y *vs.* TFY means log_2_ (C20Y/TFY). It is the expression level (RPKM) of resistant line GT-C20 compared with the susceptible line Tifrunner under *Aspergillus* infected condition (Y). The values are shaded red if ≥2 and shaded yellow if the values are ≥1.5 and <2. This applies to Table 4, Table 5, Table 6, and Table 7. The negative values are shaded green if ≤ −2 and shaded dark green if the values are ≤−1.5 and >−2.

**Table 4:** Resistant genes constitutively expressed.

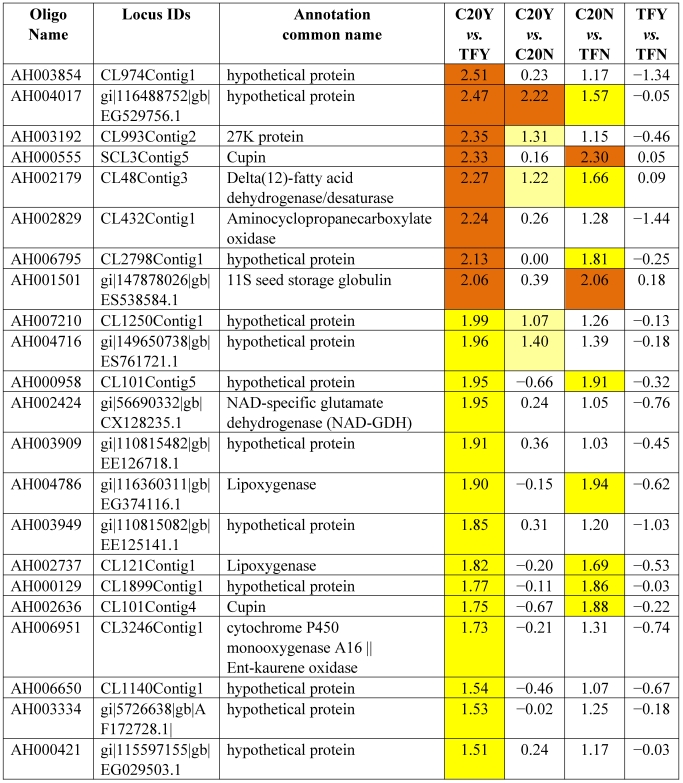

**Table 5:** Consistently down expressed genes in response to fungal infection.

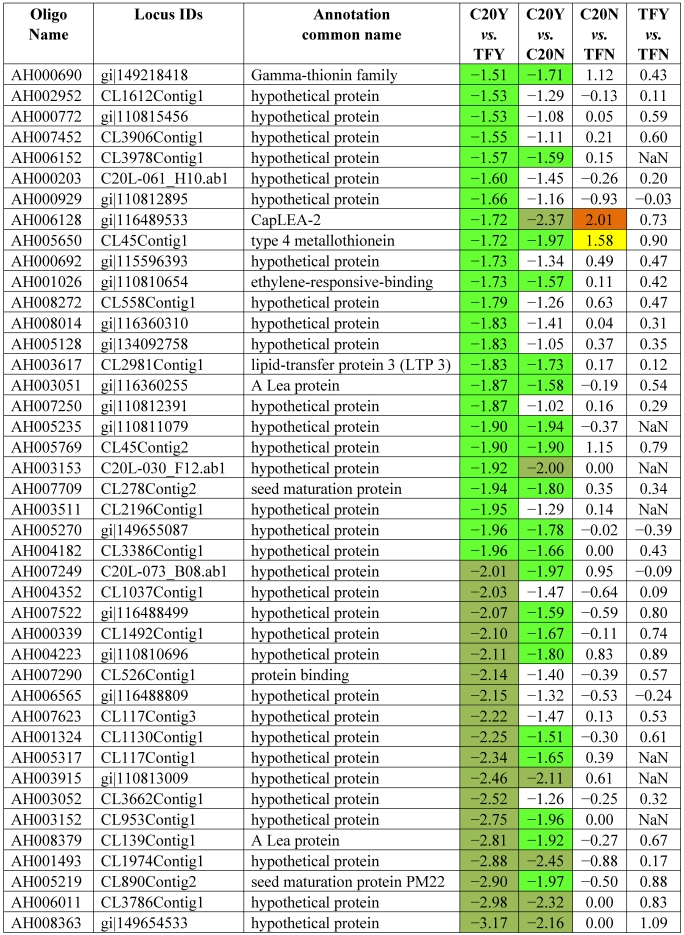

**Table 6:** Constitutively down expressed genes.

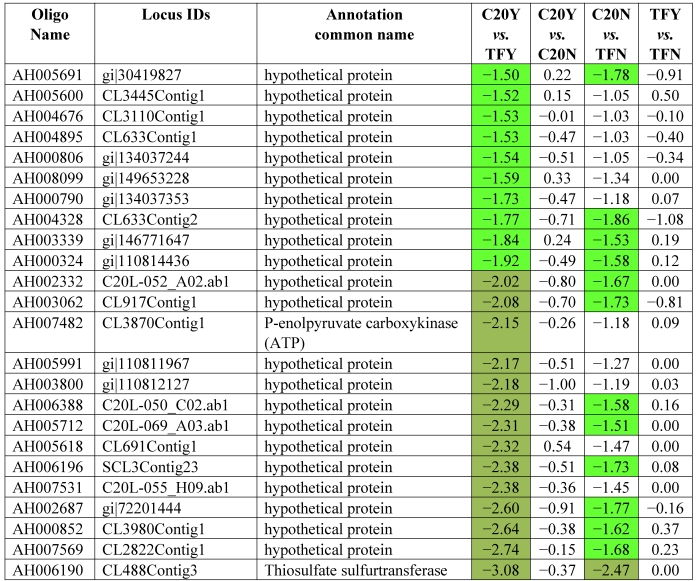

**Table 7:** GO biological processes of differentially expressed genes related to resistance.

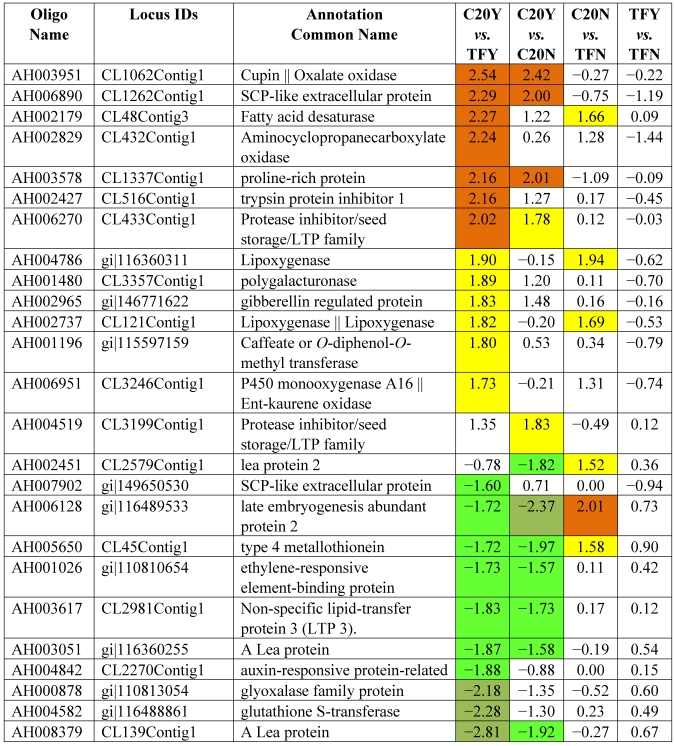

### 3.3. Genes Resistant to Fungal Infection in Other Crop Systems have been Identified

Among the genes whose putative biological functions have been postulated, we identified quite a few genes that were reportedly showing resistant to *Aspergillus* infection in other crop systems (Table 7). The trypsin protein inhibitor 1 (CL516Contig1) was demonstrated to be resistant to *A. flavus* infection in corn [24,25,26,27]. The lipoxygenase (CL121Contig1) also showed anti-fungal activities in peanut, corn, and soybean [28,29,30,31]. Several lines of evidence have indicated that lipoxygenase enzymes and their products, especially 9S- and 13S-hydroperoxy fatty acids, could play a role in the *Aspergillus*/seed interaction. Both hydroperoxides exhibit sporogenic effects on *Aspergillus* spp. and differentially modulate aflatoxin pathway gene transcription. 

Previous studies through gene cloning and characterization reported [28,29,30,31,32] the role of seed lipoxygenases, a peanut seed gene, PnLOX1. Analysis of its nucleotide sequence suggests that PnLOX1 encodes a predicted 98 kDa protein highly similar in sequence and biochemical properties to soybean LOX2. The full-length PnLOX1 cDNA was subcloned into an expression vector to determine the type(s) of hydroperoxide products that the enzyme produces. Analysis of the oxidation products of PnLOX1 revealed that it produced a mixture of 30% 9S-HPODE (9S-hydroperoxy-10E, 12Z-octadecadienoic acid) and 70% 13S-HPODE (13S-hydroperoxy-9Z, 11E-octadecadienoic acid) at pH 7. PnLOX1 is an organ-specific gene which is constitutively expressed in immature cotyledons but is highly induced by methyl jasmonate, wounding, and *Aspergillus* infections in mature cotyledons. Examination of HPODE production in infected cotyledons suggests PnLOX1 expression may lead to an increase in 9S- HPODE in the seed [28,29,30,31,32]. The human lipoxigenase was also reported to degrade aflatoxin B1 by oxidative metabolism [33,34]. Those genes demonstrating resistance to fungal infection in other crops such as corn and soybean were also identified in peanut through this microarray gene profiling experiment. This result indicates that our data are consistent with previous studies in other crops and that this study provides new evidence for the roles of these proteins in protection against fungal infection.

### 3.4. Defense-Related Genes Identified by Peanut Seed EST Database Search

The EST sequences from “GT-C20” and “Tifrunner” were compared individually against peanut seed EST database. Among the EST sequences with *R* > 4 [35], only three up-regulated putative defense-related genes were identified in both “GT-C20” and “Tifrunner” seed libraries. They were putative desiccation-related protein PCC13-62 precursor, serine protease inhibitor, and seed maturation protein LEA 4. Six up-regulated EST sequences were observed only in “GT-C20” seed EST libraries, and matched previous reported known proteins including PR10 protein, defensin protein, and calmodulin. In the “Tifrunner” seed EST libraries, five defense-related genes such as metallothionein-like protein, heat shock protein and Cu/Zn superoxide dismutase II exhibited significant up-regulation. 

In the microarray experiments, several of the late embryo abundant (LEA) or late embryogenesis protein (LEA proteins) (CL2579Contig1 and CL139Contig1) were demonstrated highly or moderately down-expressed during fungal infection. The growth hormone genes for ethylene and auxin-responsive proteins (CL2270Contig1) were also down-expressed upon fungal infection. It is interesting to find that one of the SCP-like extracellular proteins was up-expressed (CL1262Contig1) while the other SCP-like extracellular proteins were down-expressed (ES761513.1|ES761513). The mechanisms of their expression in response to fungal infection deserves further investigation.

## 4. Conclusions

We described the sequence and assembly of 13,879 unique peanut ESTs, designed and constructed a 6932 gene-element oligonucleotide microarray, and analyzed the results of gene screening on the resistant genes in peanut in response to *Aspergillus* infection. More importantly, we identified resistant genes that are highly expressed in response to fungal infection. These genes could be valuable resources for follow-on research to transfer genes into commercial peanut cultivars through conventional breeding, marker assisted breeding, or through gene transfer by biotechnology. In addition, genetic regulation may be employed to boost the expression levels of these genes in the commercial cultivars to reduce or prevent aflatoxin contamination in peanut crop. EST and microarray technology has been demonstrated as robust in screening and identifying resistant or susceptible genes in large scale if not at the genome scale. Due to the lack of peanut whole genome sequence progress, the majority of the ESTs encode hypothetical proteins with unknown functions. We here demonstrate that using EST sequences and microarray strategies to screen and profile resistance genes provides a robust approach for identifying resistance genes and resistance gene candidates in the absence of a peanut genome sequence. Data presented in this report significantly identified gene targets for future crop improvement manipulation. Both the research methods and the resulting data will prove useful in crop improvement and aflatoxin contamination prevention. 
